# Geospatial determinants of health: A global surgery lens

**DOI:** 10.4102/jcmsa.v2i1.49

**Published:** 2024-08-29

**Authors:** Moses Isiagi, Yvan Zolo, Salome Maswime

**Affiliations:** 1 Global Surgery Division, Department of Surgery, Faculty of Health Sciences, University of Cape Town, Cape Town, South Africa; 2 WHO Collaborating Center on Integrated Clinical Care, Faculty of Health Sciences, University of Cape Town, South Africa

**Keywords:** global surgery, geospatial determinants of health, surgical care, geospatial mapping

## Abstract

Global surgery represents an emerging interdisciplinary field dedicated to enhancing health outcomes and promoting health equity by addressing the unmet need for surgical, obstetric and anaesthesia care, especially among underserved populations and those facing crises. This field focusses on improving access to equitable and comprehensive surgical care and strengthening healthcare delivery systems. The Lancet Commission on Global surgery of 2015 emphasises the importance of safe, timely, affordable and universal access to surgical care, highlighting the ease of reaching a surgical provider and receiving prompt and appropriate care as crucial components of timely access. Geospatial mapping and analysis are being used to assess access to surgical care in low- and middle-income countries and Africa. This data reveal disparities in access and is crucial for understanding health outcomes in different areas. Addressing geospatial determinants of health (GDOH) is essential for improving health equity, especially in these regions. Leveraging geospatial analysis can help mitigate delays in accessing care and improve resource allocation to strengthen healthcare systems. We propose that the interrogation of GDOH is critical in advancing surgical health, averting disease and implementing surgical interventions and programmes.

## Introduction

Global surgery is a relatively new and emerging interdisciplinary field of enquiry, research, practice and advocacy. It aims to improve health outcomes and achieve health equity for all people who need surgical, obstetric and anaesthesia care, with a special emphasis on underserved, marginalised populations and those in crisis.^1^

As an emerging discipline and science, global surgery interrogates the unmet need for access to care and the quality of surgical care, focussing on strengthening the health system.^1^ Global surgery is about finding solutions to (1) access to equitable surgical care and (2) access to comprehensive transdisciplinary surgical care. Solutions can strengthen surgical healthcare delivery systems.^1^

The Lancet Commission on Global Surgery of 2015 underscores safe, timely, affordable and universal access to surgical care. Two cardinal components of timely access include the ease of reaching a surgical provider and the provision of prompt and/or appropriate care from the provider.^1^

Geospatial mapping and/or analysis (a visualisation methodology) has been used in low- and middle-income countries (LMICs) and Africa in various ways.^2,3,4^ For instance, it has been used to estimate the ease of reaching a surgical provider and the provision of prompt and/or appropriate care from the provider.^5^

In Somaliland, approximately 16.9% of the population live within a 2-h driving distance of a surgeon; in Uganda, Rwanda and Liberia, patients travel 30 km to see a surgeon, and in Ethiopia the patients travel 144 km.^5^ These findings underscore the need for more efforts to study geospatial determinants of health, as spaces and places shape and influence our lives and livelihood throughout our lifetimes.^6^ The places we inhabit daily influence our experience with disease and well-being, including being barriers or enablers.^6^

Africa is broad and diverse. The determinants of access to health and well-being vary widely by place and time, and the inclusive term for this variance is geospatial determinants of health (GDOH). Geospatial determinants of health includes ‘eco-anxiety’, which is the chronic fear of environmental factors that are currently being experienced and will likely impact a community’s healthcare, access and provision.^6^

In Africa, 1 billion people lack access to safe and timely surgery. The GDOH and its methodology may offer a key to reducing the unmet need for surgical services in LMICs.^1^

The majority of the settings in Africa have fragile and poor infrastructure, deficient health systems underscored by ineffective equipment, and a scarcity of supplies including drugs, anaesthesia and oxygen, which is further compounded by poor and/or barely existent referral systems leading to ineptitude in tracking routine procedures both in theatre and pre- and post-theatre.^7^ The multiple barriers in LMICs contribute to high postoperative mortality.^7^ Therefore, global surgery and GDOH must be explored using spatial patterns and processes, especially in the African context.

## The geospatial determinants of health

The GDOH encompass a wide range of geographic and spatial factors that significantly influence human health and well-being.^6^ The determinants include:

- *Built environment*: It is human-made or modified and encompasses buildings, residential addresses, transit stops, air and water quality, pollution exposure, climates and natural disasters. A planned built environment enables access to healthcare,^6^ and studies have shown that one’s postal code may be more predictive of health outcomes than a genetic code as a determinant of life expectancy.^8^
- *Health policy*: Includes policies instituted by governments at every level and this translates into place-based policies that target poverty alleviation, education, employment and access to comprehensive healthcare.
- *Population connectivity environment*: Includes settlements, behaviour, connections and interactions with people coupled with animal populations, intensifying or prolonging disease transmission.^6^
- *Healthcare access*: Includes adjacency to healthcare facilities, surgeons and healthcare resources.^6^

We cannot afford to overlook the importance of understanding the GDOH and global surgery. This understanding is crucial in identifying and comprehending the relationship between these determinants and health outcomes. It will guide us in targeting interventions and policy decisions to improve health equity outcomes, especially in LMICs and Africa, and prompt us to take immediate action.

Geospatial determinants of health concepts, including spatial analysis, will equip global surgery experts and public health scientists in Africa and LMICs to examine, characterise and analyse the vital relationship between healthcare facility location and healthcare access and outcomes. Donabedian’s triad (structure, process and outcomes) in the quality-improvement literature highlights that variance of healthcare quality and access only accounts for 20% of health outcomes; what accounts for everything else is what we call the social and GDOHs.^9^ Therefore, where one’s life is a massive part of that, these inform the following research priorities and questions:

1. What is the geographical access on the African continent, and what services are available in Africa?
2. What is the disease burden and outcomes in the diverse African geographical area?
3. What interventions can be implemented to strengthen the health systems in the diverse geographical area of Africa?

Geospatial determinants of health methodology, as an integral part of delivering health, should not be confined to academic circles. It should be actively integrated into public institutions. This integration is not just a recommendation, but a call to action for the global surgery community to take up the responsibility of advocating for the use of geospatial determinants in public health. In the African context, GDOH can be helpful in:

- ***Mapping the healthcare facilities in Africa***. A study on geospatial mapping of essential surgery in sub-Saharan Africa assesses accessibility and burden of surgical disease in Africa and recommends a further in-depth health facility assessment that is required to define timely and safe affordable care.^7^ This study highlights how geospatial determinants can be used to improve disease surveillance and outbreak response and explores urban planning and infrastructure development’s roles in addressing GDOH. Hardcastle et al., in South Africa, employed geospatial analysis to delineate the relative gravity of the trauma burden and underscored the potential incorporation of geospatial mapping in the development of an effective prehospital care system with an Afrocentric perspective.^10^
- ***Mapping health outcomes and identifying certain diseases and trends in Africa***. A study on spatial access to emergency services in LMICs^11^ revealed that geographic information system (GIS)-based studies are increasing in LMICs because of the ubiquity of high-quality spatial data. It demonstrated the importance and utility of geocoded data in LMICs and suggested that geospatial-based methods could be valuable in trauma system development to help direct infrastructure expansion.
- ***Addressing key indicators of the Lancet Commission on Surgery to assess and track the progress of access to surgical services and outcomes recommendations for tracking in the African continent.*** The last indicator in ‘geographical access’ highlights a need to access and track the progress of access to surgical services and outcomes.^12^ The geospatial analysis methods recommended will, in turn, address health disparities.

## Conclusion

Beyond LMICs and Africa, geospatial analysis science can help to facilitate and reduce barriers associated with geographical accessibility to surgical care. Understanding the geographical barriers to accessing care within specific time frames allows scientists, stakeholders, investors and governments to use evidence-based methods to improve the delivery of surgical services for vulnerable and underserved populations. Geospatial analysis and GDOH primarily addresses the delay in ‘reaching’ the health facility and, to a certain extent, the delay in ‘receiving’ appropriate care.^9^
